# AKI Subtyping and Prognostic Analysis Based on Serum Electrolyte Features in ICU

**DOI:** 10.3390/jcm14217623

**Published:** 2025-10-27

**Authors:** Wentie Liu, Tongyue Shi, Haowei Xu, Huiying Zhao, Guilan Kong

**Affiliations:** 1 Institute of Medical Technology, Peking University Health Science Center, Beijing 100191, China; 2211210777@stu.pku.edu.cn (W.L.);; 2 National Institute of Health Data Science, Peking University, Beijing 100191, China; haoweixu0126@163.com; 3 Advanced Institute of Information Technology, Peking University, Hangzhou 311215, China; 4 Department of Critical Care Medicine, Peking University People’s Hospital, Beijing 100044, China; zhaohuiying109@sina.com

**Keywords:** Acute Kidney Injury, serum electrolytes, subtype identification, Intensive Care Unit, K-Medoids clustering

## Abstract

**Objective**: To identify distinct subtypes of ICU patients with Acute Kidney Injury (AKI) using serum electrolyte data and assess their associations with in-hospital mortality risk. **Methods**: This study used the eICU Collaborative Research Database (eICU-CRD) as its primary data source. AKI patients were identified according to the KDIGO clinical practice guidelines. Using K-Medoids clustering, we identified distinct AKI subtypes based on the first serum electrolyte measurements taken within 24 h of AKI diagnosis in the ICU. Logistic regression analysis was then employed to assess associations between these subtypes and in-hospital mortality risk. Within each subtype, we further examined the relationship between two AKI-related treatments, diuretics and renal replacement therapy (RRT), and mortality risk. Finally, to validate the identified subtypes, we replicated the entire analysis using a critical care dataset from a grade A tertiary hospital in Beijing, China. **Results**: We identified three distinct AKI subtypes from 15,838 eligible patients in the eICU-CRD. Subtype 1 (6364 patients, 40.2%) showed the lowest risk of in-hospital death and had all serum electrolyte levels within normal ranges. Subtype 2 (6624 patients, 41.8%) carried a moderate death risk and was characterized by abnormally high chloride levels. Subtype 3 (2850 patients, 18.0%) had the highest death risk, presenting with high serum phosphate and low bicarbonate levels. Importantly, the associations between treatments and mortality risk differed significantly by subtype. In the high-risk Subtype 3, both diuretics (OR = 0.71, *p* = 0.010) and RRT (OR = 0.78, *p* = 0.045) were associated with a lower risk of in-hospital death. However, in Subtype 2, both diuretics (OR = 1.30, *p* = 0.044) and RRT (OR = 1.56, *p* = 0.003) were associated with an increased risk. Neither treatment showed a significant association with death risk in Subtype 1. These findings were validated in the critical care database (431 AKI patients) from a Chinese local hospital, where the same three subtypes emerged with consistent electrolyte patterns, death risk profiles, and associations between treatments and mortality risks, validating the stability of the identified subtypes. **Conclusions**: Serum electrolyte data can help identify ICU AKI subtypes with different mortality risks. Additionally, associations between treatments (diuretics and RRT) and mortality risk vary significantly across these subtypes. These results generate the hypothesis that AKI subtyping could potentially inform personalized management strategies.

## 1. Introduction

Acute kidney injury (AKI) is defined as a syndrome of rapid renal function decline. It is a common complication in critically ill patients and is associated with high morbidity and mortality [1,2]. AKI is linked to diverse etiologies and pathophysiological mechanisms, significantly impacting patient prognosis [3]. Its incidence and mortality rates have been rising [4]. Globally, an estimated 10–20% of hospitalized patients are affected by AKI [5,6]. A 2013 multicenter study in China, involving 44 hospitals and reviewing cases of 374,286 patients, reported an AKI incidence rate of 2.03% (7604 of 374,286) and a mortality rate of 12.4% (927 of 7475, 129 of the 7604 patients with AKI detected had missing information on mortality). Based on the results, it was estimated that there were 1.4 million adult hospital patients with AKI according to the KDIGO criteria during 2013, incurring total hospitalization costs of US$13 billion in China [7]. A European study in 2023 on the disease burden of real-world AKI patients showed that the annual treatment cost of AKI has been rising over the past 20 years, resulting in a cumulative direct cost of €329 million [8]. It is worth noting that COVID-19 might further increase this burden by elevating the risk of acute kidney disorders, especially in patients with moderate to severe illness [9]. 

The burden of AKI is even higher in Intensive Care Units (ICUs). A study across 17 ICU centers in Southeast Asia in 2020 found an AKI incidence of 52.9% (2471 out of 4668 patients) [10]. Similarly, another study in 2024 reported an AKI incidence of 53.3% and an AKI mortality rate of 42.1% in ICUs within the Amazon region [11]. These findings collectively highlight the substantial disease and economic burden posed by AKI. This burden is exacerbated by limited ICU resources, which demand efficient and patient-centric care strategies [12]. Such strategies, including telemedicine as seen in outpatient urology, aim to optimize resource allocation [13]. Crucially, personalizing care also depends on addressing the profound heterogeneity among individual patients.

AKI is a clinically heterogeneous condition. Patients can differ significantly in their underlying causes, clinical features, response to treatments, and outcomes. This variability necessitates personalized treatment approaches, a topic attracting considerable attention from clinicians and researchers in the AKI field [14].

To address AKI heterogeneity, a common strategy for personalized medicine involves grouping patients into distinct subtypes based on clinical characteristics and then tailoring treatments to each subgroup [15]. Traditional AKI classification relies on domain knowledge and expert consensus. For example, AKI is often categorized by cause and location as prerenal, intrinsic renal, or postrenal [16]. The KDIGO (Kidney Disease: Improving Global Outcomes) clinical guidelines classify AKI into stages 1, 2, and 3 based on serum creatinine levels and urine output [17]. Chaudhari et al. regarded the use of contrast agents as the cause of AKI, and proposed two subcategories, namely Contrast-Associated Acute Kidney Injury (CA-AKI) and Contrast-Induced Acute Kidney Injury (CI-AKI) [18]. CA-AKI was defined as a sudden decline in renal function within 48 h after intravenous or arterial use of iodine contrast agents, and CI-AKI was defined as a sudden decline in renal function within several days after the use of iodine contrast agents.

Advances in computing and big data have spurred data-driven approaches to identify AKI subtypes. Chaudhary et al. extracted 2546 combined features from structured electronic health record (EHR) data and applied K-Means clustering to 4001 sepsis-associated AKI patients in the MIMIC-III database, identifying three subtypes [19]. Subtype 1 had the lowest prevalence of liver disease, Subtype 2 had the highest Simplified Acute Physiology Score II (SAPS II), and Subtype 3 patients had the highest bilirubin and aspartate aminotransferase levels. Bhatraju et al. combined blood and urinary biomarkers to define AKI subtypes, finding two subtypes, with Subtype 1 having a higher rate of prior congestive heart failure, and Subtype 2 linked to adverse kidney events [20]. Similarly, based on comorbidities, baseline clinical data, and biomarkers, Wiersema et al. performed latent class analysis on 301 septic AKI patients and identified two subtypes: Subtype 1, characterized by a significantly higher body mass index (BMI), was associated with better renal recovery and a lower 90-day mortality risk. In contrast, Subtype 2, which had higher biomarker levels of HBP, Ela, PRTN3, and MMP8, was associated with impaired renal recovery and a significantly increased risk of 90-day mortality [21].

However, current research on AKI subtyping has limitations. First, domain knowledge-based approaches are constrained by the existing clinical guidelines, failing to effectively utilize the rich, high-dimensional clinical data generated in practice. Second, data-driven subtyping studies may incorporate an excessive number of clinical and laboratory variables. The high dimensionality of real-world data in subtyping analysis increases computational complexity, brings difficulties in real-time data accessibility, and conflicts with the need for timely clinical decisions in critical care settings like ICUs.

Electrolyte imbalances are strongly linked to AKI. They are established risk factors for AKI development [22] and mortality in AKI patients [23]. For example, severe hyperkalemia can induce renal tubular necrosis, triggering AKI [24]. Meanwhile, AKI itself impairs water, sodium, and metabolite regulation, leading to electrolyte disorders. In the existing data-driven AKI subtype studies, serum electrolytes were also included as part of the clustering variables [25,26,27]. Among them, Tan et al. identified three subtypes based on a total of 22 laboratory indicators, including six serum electrolytes: sodium, potassium, chloride, phosphate, calcium, and magnesium [27]. They found that Subtype 1 had the highest serum chloride; Subtype 2 had the highest serum sodium; and Subtype 3 had the highest serum phosphate. Serum electrolytes have also been used as exclusive clustering variables in critical care subtyping research. Xiao et al. clustered all ICU patients using only seven electrolytes, including sodium, potassium, calcium, magnesium, phosphate, chloride, and bicarbonate, and found three different electrolyte disorder subtypes closely related to prognosis in critically ill patients [28]. Their analysis results revealed that Subtype 1 had significant kidney injury, Subtype 2 was more severe in the nervous system, and Subtype 3 had the best prognosis. By analogy, serum electrolyte profiles could be used to identify AKI subtypes in critically ill patients.

In this study, we applied K-Medoids clustering to identify distinct AKI subtypes by leveraging the first serum electrolyte measurements taken within 24 h of ICU AKI diagnosis. In addition, we aimed to explore the subtype-in-hospital mortality association and the association between treatments and in-hospital mortality within each AKI subtype.

## 2. Materials and Methods

### 2.1. Database

The eICU Collaborative Research Database (eICU-CRD) is a public resource, developed jointly by Philips Healthcare and MIT’s Laboratory for Computational Physiology (LCP) [29]. This multicenter dataset captures critical care information from multiple hospitals across the United States, specifically including 208 hospitals with 200,859 ICU admissions recorded between 2014 and 2015. The eICU-CRD was used as the primary database for AKI subtyping analysis.

Also, we constructed a critical care database based on ICU patient data recorded in the Electronic Medical Record (EMR) system from a grade A tertiary hospital in China. It contains records from 2022 to 2024, capturing demographic characteristics and laboratory test results documented during patient encounters. This database served as a validation cohort to confirm the stability of AKI subtypes identified in eICU-CRD. All patient identifiers in this dataset were removed through de-identification procedures.

### 2.2. Patients

We identified AKI patients using the 2012 KDIGO clinical guideline. AKI was defined based on changes in serum creatinine (SCr) or urine output. Patients meeting any of the following criteria were identified as having AKI: (1) SCr increases ≥0.3 mg/dL (≥26.5 μmol/L) within 48 h; (2) SCr rise to ≥1.5 times baseline within 7 days; (3) Urine output <0.5 mL/kg/h sustained for ≥6 h. The baseline definition of SCr is the lowest measurement of creatinine within 7 days after the patient is admitted to the ICU.

AKI patients who met all the following criteria were included for analysis: (1) aged > 18 years; (2) met the KDIGO AKI definition above; (3) AKI diagnosis occurred during their ICU stay; (4) all target electrolyte measurements were available within 24 h of AKI diagnosis.

Patients were excluded if any of the target electrolyte measurements were missing within 24 h after the diagnosis of AKI.

Due to the small number of patients and the low rate of electrolyte data absence in the Chinese local critical care database, mean imputation was performed on the electrolytes with missing data.

### 2.3. Variables

We selected seven serum electrolytes that are frequently tested in clinical practice and closely related to AKI as variables for clustering analysis. During data preprocessing, it was found that the missing rate of serum calcium exceeded 30%. Therefore, only six routinely measured serum electrolytes were finally used as clustering variables: sodium, potassium, chloride, phosphate, magnesium, and bicarbonate (all are continuous variables). The first measurements taken within 24 h of AKI diagnosis were used for clustering analysis.

For the analysis of associations between AKI subtypes and the risk of in-hospital mortality, and associations between treatments and in-hospital mortality within different subtypes, we adjusted for potential confounders. Covariates were chosen by referring to prior AKI prognostic analysis literature and considering clinical relevance, data availability, and inclusion in critical illness severity scores such as Acute Physiology and Chronic Health Evaluation-II (APACHE-II). The final selected confounders included: gender, age, heart rate, respiratory rate, hemoglobin, white blood cell count, platelet count, blood urea nitrogen (BUN), and blood glucose. Covariate data were extracted at the same time point as the serum electrolytes.

### 2.4. Methods

We employed K-Medoids clustering for subtype identification. The K-Medoids algorithm was selected for clustering due to its robustness to outliers, a critical advantage when clustering clinical electrolyte data that may contain extreme values. K-Medoids clustering works by selecting actual data points (medoids) as cluster centers and iteratively swapping them to minimize the total dissimilarity within clusters, resulting in more stable partitions [30].

To determine the optimal cluster number (*k*), we used the elbow method and Davies–Bouldin (DB) index. The elbow method plots the Within-Cluster Sum of Squares (WCSS) against the number of clusters. While WCSS decreases with increasing *k*, the rate of decrease slows markedly at a distinct inflection point (“elbow”), indicating the optimal *k*. Meanwhile, a lower DB score reflects better clustering quality, signaling tighter clusters with greater separation between them.

Next, we used logistic regression analysis to assess the association between AKI subtypes and in-hospital mortality. In this model, the identified AKI subtypes served as the independent variable, with in-hospital mortality as the outcome.

We then performed analysis within each AKI subtype to explore the association between treatment strategies and mortality risk. Specifically, we analyzed two common interventions: diuretics and renal replacement therapy (RRT).

All data preprocessing and clustering were performed using Python 3.9 and SPSS 23.0. Statistical significance was set at *p* < 0.05.

## 3. Results

### 3.1. Baseline Data Characteristics of AKI Patients

A total of 15,838 AKI patients were included in the primary dataset eICU-CRD, and 431 AKI patients were included in the Chinese local critical care database as the validation dataset. The baseline data characteristics of the patients in both databases are shown in Table 1.

### 3.2. AKI Subtypes

**Figure 1:**
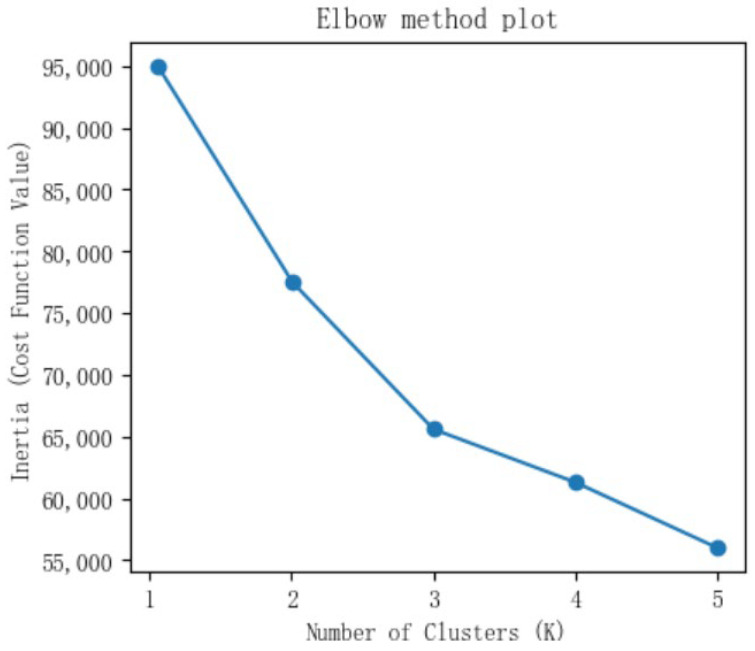
The Elbow method plot derived from AKI patient data in eICU-CRD.

**Figure 2:**
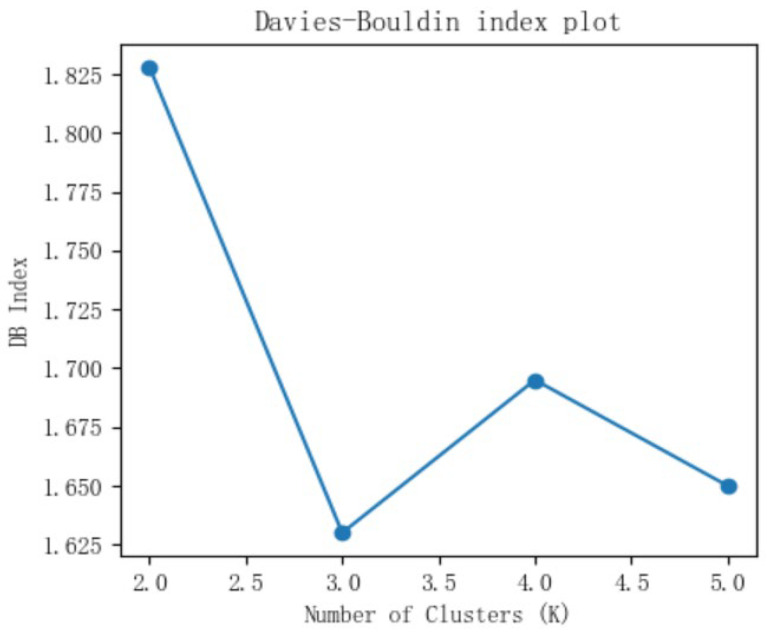
The Davies–Bouldin index plot in eICU-CRD.

The elbow method and DB index plots derived from AKI patient data in eICU-CRD are presented in Figure 1 and Figure 2, respectively. The elbow method plot shows a clear inflection point at *k* = 3, indicating 3 as the optimal number. Meanwhile, the DB index reached its minimum value at *k* = 3. Taken together, these metrics jointly determined *k* = 3 as the optimal number of clusters.

**Figure 3:**
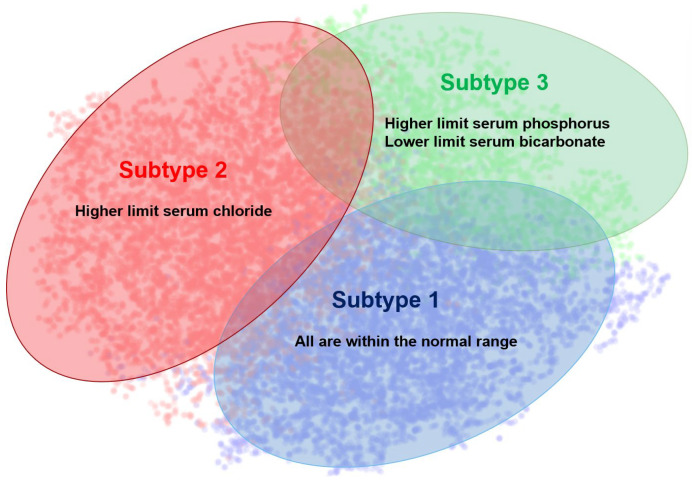
Three AKI subtypes identified from AKI patient data in eICU-CRD. (“Higher limit” and “Lower limit” represent values that exceed the upper or fall below the lower bounds of the normal range.).

Taking *k* = 3 as the parameter for clustering, we identified three distinct AKI subtypes: Subtype 1 (Normal electrolyte subtype, n = 6364, 40.2%) with all electrolytes within normal ranges; Subtype 2 (Hyperchloremic, n = 6624, 41.8%) showing elevated serum chloride; and Subtype 3 (High-phosphate and low-bicarbonate, n = 2850, 18.0%) characterized by elevated serum phosphate and low serum bicarbonate levels. Detailed electrolyte distributions are presented in Table 2, while Figure 3 visually represents this subtype distribution through t-SNE dimensionality reduction (implemented using Python 3.9’s scikit-learn and matplotlib libraries) in the eICU-CRD cohort.

Following the same optimal cluster size identification procedure as for eICU-CRD, we identified the optimal cluster number (*k* = 3) using the elbow method and DB index in the Chinese local critical care database. Applying *k* = 3, we also identified three AKI subtypes: Subtype 1 (Normal electrolyte subtype, n = 117, 27.1%), Subtype 2 (Hyperchloremic, n = 245, 56.8%), and Subtype 3 (High-phosphate and low-bicarbonate, n = 69, 16.1%). All three subtypes maintained consistent clinical patterns across cohorts. Subtype 1 showed the lowest in-hospital mortality, Subtype 2 exhibited hyperchloremia, and Subtype 3 demonstrated hyperphosphatemia with low bicarbonate. However, Subtype 1 in the validation cohort displayed mild hyponatremia with normal levels of other electrolytes, a feature not observed in the eICU-CRD cohort. The elbow method plot and the DB index plot are shown in Figures S1 and S2 of the Supplementary Materials. Relevant electrolyte distribution tables and dimension reduction diagrams are provided in Table S1 and Figure S3 of the Supplementary Materials.

### 3.3. Prognosis Analysis of AKI Subtypes

As shown in Table 3, both Subtype 2 (Hyperchloremic, OR = 1.13, *p* = 0.025) and Subtype 3 (High-phosphate and low-bicarbonate, OR = 1.52, *p* < 0.001) had significantly higher in-hospital mortality risk compared to Subtype 1 (Normal electrolyte subtype). Also, Subtype 3 carried a higher mortality risk than Subtype 2 (OR = 1.43, *p* < 0.001).

Table 4 summarizes the association between treatments and mortality risk within each subtype. In Subtype 1 (Normal electrolyte subtype), neither the use of diuretics nor RRT showed a significant association with in-hospital mortality. For Subtype 2 (Hyperchloremic), both diuretics (OR = 1.30, *p* = 0.044) and RRT (OR = 1.56, *p* = 0.003) had associations with increased mortality risk. Notably, in Subtype 3 (High-phosphate and low-bicarbonate), both treatments were associated with reduced mortality risk (Diuretics: OR = 0.71, *p* = 0.010; RRT: OR = 0.78, *p* = 0.045).

Replicating the same association analysis in the Chinese local critical care database confirmed identical patterns: Subtype 3 had the highest in-hospital mortality risk, followed by Subtype 2, and Subtype 1 showed the lowest risk. Meanwhile, the treatment-mortality relationships were also similar to the eICU-CRD cohort, with no significant association in Subtype 1, diuretics or RRT associated with increased mortality risk in Subtype 2, and consistent mortality risk reduction by both treatments in Subtype 3. Detailed association analysis results are available in Tables S2 and S3 of the Supplementary Materials.

## 4. Discussion

Using the multicenter ICU dataset eICU-CRD, we performed exploratory K-Medoids clustering on 15,838 ICU patients with AKI based on their first serum electrolyte measurements within 24 h of AKI diagnosis. This analysis identified three distinct AKI subtypes characterized by unique electrolyte patterns. The Subtype 2 (Hyperchloremic) and Subtype 3 (High-phosphate and low-bicarbonate) had significantly higher in-hospital mortality risk compared to Subtype 1 (Normal electrolyte), which had normal electrolyte levels. Logistic regression analysis showed that these subtypes had significantly different risks of in-hospital mortality. We further examined the associations between treatments and mortality risk across different subtypes and found that neither treatment showed a significant association with death risk in Subtype 1; both diuretics and RRT were associated with an increased risk in Subtype 2, and both diuretics and RRT were associated with a lower risk of in-hospital death in Subtype 3. Finally, we validated the identified subtypes in a separate cohort of 431 AKI patients from a Chinese local critical care database, using identical variables and analytic methods. Consistent AKI subtype patterns emerged across both datasets.

Compared with Subtype 2 and Subtype 1, Subtype 3 had a higher risk of in-hospital mortality. It had disorders of two electrolytes, namely higher serum phosphate and lower serum bicarbonate. Phosphate dysregulation is critical in AKI. A randomized controlled trial revealed that AKI patients with hyperphosphatemia had a higher 90-day mortality [31]. In patients with AKI, the deficiency of bicarbonate can cause serious adverse consequences. Lee et al. found that the more severe the metabolic acidosis of patients was, the worse the outcomes were, such as mortality [32]. Consequently, Subtype 3, marked by hyperphosphatemia and low bicarbonate, showed the highest mortality risk in this study.

Regarding Subtype 2, its mortality risk was higher than Subtype 1, which had normal electrolytes. The most notable feature of Subtype 2 was high serum chloride levels, exceeding the normal range. High serum chloride intensifies the inhibition of renin and angiotensin II, leading to renal vasoconstriction and reduced glomerular filtration, and further damaging the renal function of patients [33]. Previous studies have established hyperchloremia as a mortality risk factor in critically ill ICU patients [34,35]. Also, we observed higher mortality in hyperchloremic AKI patients compared to patients with normal electrolytes in this study.

Subtype 1 was consistently associated with the lowest in-hospital mortality in both cohorts. While mild hyponatremia was observed in the validation cohort, it was not present in the discovery cohort. This suggests that hyponatremia may not substantially influence in-hospital mortality, which is consistent with findings in the literature [36].

Additionally, our study reveals significant variation in the association between treatments and in-hospital mortality in different AKI subtypes, demonstrating AKI’s clinical heterogeneity. A systematic review summarizing current knowledge on AKI subtypes driven by clinical and biomarker data also suggested that subtype identification might enable targeted therapies [37]. In the literature, studies exploring the association between diuretics, RRT, and outcomes like death in AKI patients have produced inconsistent conclusions. About diuretics, Zhao et al. [38] analyzed 14,154 AKI patients using the MIMIC-III database through Cox proportional hazards modeling and observed that diuretics was associated with a lower risk of in-hospital mortality. In a study of 1663 patients with sepsis-associated acute kidney injury (SA-AKI) from the MIMIC-IV database, Li et al. [39] applied logistic regression analysis and identified significantly lower in-hospital mortality risk among those receiving diuretic therapy. However, after studying 552 AKI patients in 4 ICUs affiliated with the University of California, Mehta et al. found that diuretics increased the risk of death [40]. Meanwhile, Shigehiko et al. evaluated diuretics in 1743 AKI patients across ICUs in 23 countries and found no significant link between diuretics and mortality [41]. In a pilot randomized blinded controlled trial across three ICUs, Bagshaw et al. enrolled 73 AKI patients and found that early furosemide infusion did not significantly reduce in-hospital mortality risk [42]. Regarding RRT, the KDIGO guidelines recommend RRT for specific problems like metabolic acidosis or high potassium levels [17]. But some studies question its benefit. A logistic regression analysis of 2846 patients with AKI based on the OUTCOMEREA database showed that a higher mortality in AKI patients receiving RRT compared to those without it [43]. Metnitz et al. studied 17,126 patients admitted to Austrian ICUs and found that patients receiving RRT for AKI had a higher risk of dying in the hospital [44].

Some studies suggest that illness severity should be taken into account in exploring the association between treatments and in-hospital mortality among AKI patients [38,45,46,47]. Zhao et al. [38] found that furosemide worked well for patients in AKI UO stage 2–3 (AKI stages 2–3 based on urine output criteria), but not for those in AKI SCr stage 2–3 (AKI stages 2–3 based on serum creatinine criteria). Shen et al. [45] found no mortality association with continuous furosemide infusion in their meta-analysis of loop diuretics in AKI patients. However, after excluding three mild-disease studies, the pooled results showed that continuous infusion was associated with lower mortality. They concluded that treatment response to diuretics varies by disease severity. Wilson et al. studied the association between RRT and in-hospital mortality in 6119 AKI patients, and their results showed that SCr levels had an impact on this relationship [46]. Specifically, starting dialysis was linked to better survival in AKI patients with higher SCr levels, but starting dialysis was associated with an increased risk of death in patients with lower creatinine concentrations. Libório et al. studied 10,245 AKI patients and found that RRT was not linked to lower mortalities [47]. However, when including complications like metabolic acidosis, high potassium, or fluid overload in their analysis, RRT then showed a link to better hospital survival.

The above findings match our findings about the identified AKI subtypes to some extent. In our study, Subtype 3 with metabolic acidosis had the highest mortality risk; both RRT and diuretics showed a negative association with patient mortality in this patient group. Subtype 2 had intermediate mortality risk characterized by hyperchloremia and positive treatment-mortality correlations for both interventions. Subtype 1 consistently showed the lowest mortality and no significant treatment-mortality association in both cohorts. The mild hyponatremia observed in the validation cohort does not affect this conclusion.

Therefore, selecting the appropriate treatment is important for AKI patient management. Whether diuretics or RRT are suitable for an individual patient requires careful assessment of their specific characteristics.

The advantages of this study are its clinical explanation and applicability. The electrolyte-based AKI subtypes identified in this study demonstrated clinically meaningful and explainable electrolyte profiles, mortality risks, and treatment-mortality associations. Clinical applicability stems from electrolytes being routine ICU tests with timely results and low cost, which are critical for acute care decision-making.

However, several limitations exist. First, our analysis was constrained by the dataset content, as not all relevant electrolytes could be included. Specifically, serum calcium had a high rate of missing data and was excluded from analysis, potentially limiting the comprehensiveness and precision of subtype analysis. Second, our study shares the fundamental limitation of all observational research. The identified associations between treatments and outcomes are susceptible to confounding, particularly by indication (e.g., the decision to initiate RRT is inherently linked to underlying disease severity), and therefore cannot be interpreted as causal relationships. Third, reliance on cross-sectional electrolyte data means that fluctuations in electrolyte data over time have not been captured. As AKI electrolyte levels often vary with disease progression, using a single measurement may miss dynamic patterns that are crucial for understanding AKI progression. In the future, a longitudinal analysis of serial electrolyte measurements is recommended to capture the temporal evolution of electrolyte disturbances and to explore the dynamic effects of treatments on patient outcomes over time.

## 5. Conclusions

Using the first serum electrolyte measurements within 24 h following AKI diagnosis in the ICU, three clinically meaningful and interpretable AKI subtypes were identified and later validated in a third-party database. The identified three AKI subtypes exhibited distinct electrolyte profiles, had differential in-hospital mortality risks. The associations between treatments and outcomes identified in this study are derived from observational data and do not imply causality due to potential confounding. Collectively, these findings generate the hypothesis that subtyping based on initial electrolyte patterns may inform personalized management strategies for AKI in critical care settings, but this approach requires validation in prospective longitudinal data analysis that accounts for the dynamic nature of AKI.

## Figures and Tables

**Table 1:** Baseline data characteristics of AKI patients.

Variables	Mean Value (±Standard Deviation)/ Frequency (Percentage)	
	eICU-CRD (n= 15,838)	Chinese Local Critical Care Database (n = 431)
Age	62.22 ± 16.29	68.80 ± 8.11
Male, n (%)	8654 (54.64)	239 (55.45)
Heart rate, BPM	119.88 ± 24.73	103.89 ± 33.63
Respiratory rate, breaths/min	33.51 ± 9.70	35.33 ± 16.90
Blood glucose, mg/dL	153.71 ± 88.78	132.40 ± 25.23
Platelets, 10^9^/L	194.52 ± 106.02	150.22 ± 62.65
White blood cells, 10^9^/L	12.88 ± 9.50	14.51 ± 8.32
Blood urea nitrogen, μmol/L	24.21 ± 11.14	27.68 ± 12.92
Hemoglobin, g/dL	10.38 ± 2.01	10.41 ± 3.88
Diuretics, n (%)	1381 (8.72)	109 (25.29)
RRT, n (%)	1128 (7.12)	74 (17.17)
In-hospital mortality, n (%)	2748 (17.35)	158 (36.66)

**Table 2:** The distribution of serum electrolytes among the three AKI subtypes in eICU-CRD.

Variables (mmol/L)	Mean Value (±Standard Deviation)		
	Subtype 1 (n = 6364)	Subtype 2 (n = 6624)	Subtype 3 (n = 2850)
Serum Sodium	136.26 ± 4.63	142.95 ± 4.75	136.24 ± 5.42
Serum Potassium	3.96 ± 0.52	3.89 ± 0.52	5.01 ± 0.81
Serum Chloride	100.48 ± 4.95	110.89 ± 4.73	102.51 ± 6.72
Serum Phosphate	1.04 ± 0.33	0.98 ± 0.35	1.88 ± 0.67
Serum Magnesium	0.79 ± 0.14	0.79 ± 0.16	0.90 ± 0.22
Serum Bicarbonate	26.68 ± 4.91	22.03 ± 4.20	19.30 ± 5.12

Normal value range (mmol/L): Sodium: 135–145; Potassium: 3.5–5.5; Chloride: 95–105; Phosphate: 0.81–1.45; Magnesium: 0.67–1.04; Bicarbonate: 22–27. The shaded values indicate that they are beyond the limits of the normal reference range.

**Table 3:** Logistic regression analysis of AKI subtypes and the risk of in-hospital mortality in eICU-CRD.

Variables	In-Hospital Mortality				In-Hospital Mortality	
	OR	95% CI	*p*	OR	95% CI	*p*
Subtype 1						
Subtype 2	1.13	1.02–1.25	0.025			
Subtype 3	1.52	1.33–1.73	<0.001	1.43	1.25–1.63	<0.001

The included covariates were age, gender, heart rate, respiratory rate, white blood cells, hemoglobin, blood urea nitrogen, platelets, and blood glucose.

**Table 4:** Association analysis of treatments and in-hospital mortality in three AKI subtypes in eICU-CRD.

Treatments	Subtype 1			Subtype 2			Subtype 3		
	*OR*	95% CI	*p*	*OR*	95% CI	*p*	*OR*	95% CI	*p*
Diuretics	1.25	0.99–1.58	0.066	1.30	1.01–1.66	0.044	0.71	0.50–0.99	0.010
RRT	1.30	0.95–1.77	0.097	1.56	1.17–2.09	0.003	0.78	0.61–0.99	0.045

The included covariates were age, gender, heart rate, respiratory rate, white blood cells, hemoglobin, blood urea nitrogen, platelets, and blood glucose.

## Data Availability

The eICU-CRD database used in the current study is available at https://eicu-crd.mit.edu. And the Chinese local critical care database remains the property of the originating institution under China’s healthcare data management regulations and cannot be made publicly available. Access requires institutional approval and compliance with national data security laws.

## Supplementary Materials

The following supporting information can be downloaded at: https://www.mdpi.com/article/10.3390/jcm14217623/s1, Figure S1: The Elbow method plot derived from AKI patient data in the Chinese local critical care database; Figure S2: The Davies–Bouldin index plot based on data in the Chinese local critical care database; Figure S3: Three AKI subtypes identified from AKI patient data in the Chinese local critical care database; Table S1: The distribution of serum electrolytes among the three AKI subtypes in the Chinese local critical care database; Table S2: Logistic regression analysis of AKI subtypes and the risk of in-hospital mortality in the Chinese local critical care database; Table S3: Association analysis of treatments and in-hospital mortality in three AKI subtypes in the Chinese local critical care database.

## Abbreviations

AKI	Acute Kidney Injury
ICU	Intensive Care Unit
KDIGO	Kidney Disease: Improving Global Outcomes
SCr	Serum Creatinine
RRT	Renal Replacement Therapy
