# Supplementary material for: AKI Subtyping and Prognostic Analysis Based on Serum Electrolyte Features in ICU
Source: J Clin Med. 2025 Oct 27;14(21):7623. doi: 10.3390/jcm14217623 (PMC12607972; doi:10.3390/jcm14217623)
Supplement: Supplementary file 1 [file jcm-14-07623-s001.zip › jcm-3849808-supplementary.pdf]

Supplementary Materials

AKI Subtyping and Prognostic Analysis Based on Serum Electrolyte Features in ICU

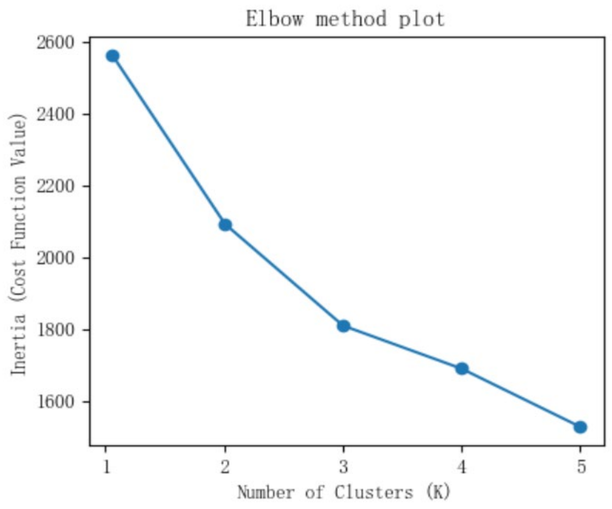

Figure S1. The Elbow method plot derived from AKI patient data in the Chinese local critical care database.

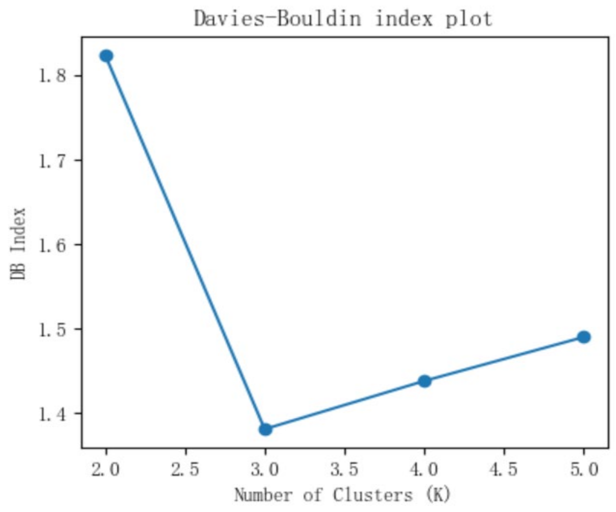

Figure S2. The Davies–Bouldin index plot based on data in the Chinese local critical care database.

Supplementary Materials

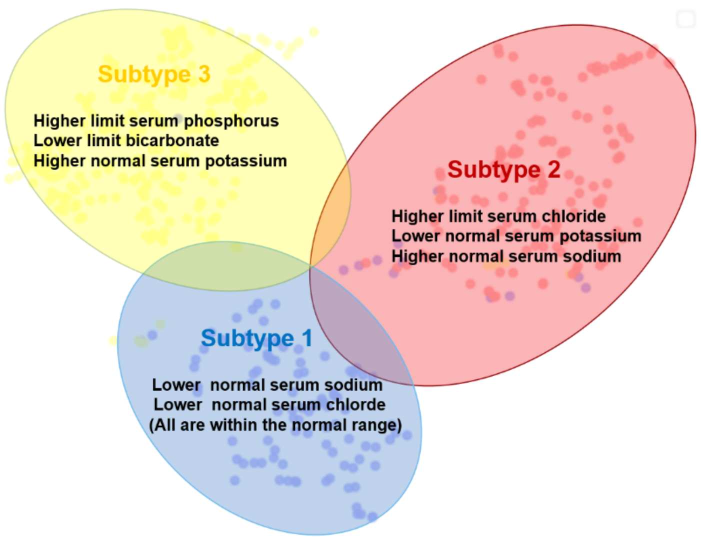

Figure S3. Three AKI subtypes identified from AKI patient data in the Chinese local critical care database. ("Higher normal" and "Lower normal" denote values that fall within the normal range but trend toward the upper or lower end, respectively. Conversely, "Higher limit" and "Lower limit" represent values that exceed the upper or fall below the lower bounds of the normal range.)

Table S1. The distribution of serum electrolytes among the three AKI subtypes in the Chinese local critical care database.

**Variables(mmol/L)**	**Mean Value (±Standard Deviation)**		
	Subtype 1 (n = 117)	Subtype 2 (n = 245)	Subtype 3 (n = 69)
Serum Sodium	133.73 ± 5.41	139.63 ± 4.57	137.28 ± 6.35
Serum Potassium	4.43 ± 0.79	3.97 ± 0.55	5.09 ± 0.96
Serum Chlorine	98.80 ± 6.18	109.09 ± 5.24	104.71 ± 6.95
Serum Phosphorous	1.22 ± 0.37	1.03 ± 0.29	2.26 ± 0.58
Serum Magnesium	0.86 ± 0.18	0.72 ± 0.16	0.94 ± 0.21
Serum Bicarbonate	26.30 ± 5.43	22.35 ± 5.05	19.20 ± 4.92

Normal value range (mmol/L): Sodium: 135-145; Potassium: 3.5-5.5; Chloride: 95-105; Phosphorus: 0.81-1.45; Magnesium: 0.67-1.04; Bicarbonate: 22-27. The shaded values indicate that they are beyond the limits of normal reference range.

Supplementary Materials

Table S2. Logistic regression analysis of AKI subtypes and the risk of in-hospital mortality in the Chinese local critical care database.

Variables	In-hospital mortality			In-hospital mortality		
	OR	95%CI	*p*	OR	95%CI	*p*
Subtype 1						
Subtype 2	2.07	1.19-3.58	0.010			
Subtype 3	2.65	1.28-5.48	0.008	2.39	1.09-5.21	0.029

The included covariates were age, gender, heart rate, respiratory rate, white blood cells, hemoglobin, blood urea nitrogen, platelets, and blood glucose.

Table S3. Association analysis of treatments and in-hospital mortality in three AKI subtypes in the Chinese local critical care database.

Treatments	Subtype 1			Subtype 2			Subtype 3		
	OR	95%*CI*	*P*	*OR*	95%*CI*	*P*	*OR*	95%*CI*	*P*
**Diuretic use**	2.29	0.87-6.04	0.094	5.36	1.50-19.14	0.010	0.02	0.01-0.54	0.021
**RRT**	1.05	0.34-3.20	0.931	12.31	4.13-36.73	<0.001	0.02	0.01-0.24	0.001

The included covariates were age, gender, heart rate, respiratory rate, white blood cells, hemoglobin, blood urea nitrogen, platelets, and blood glucose.
